# Effects of *Laminaria japonica* polysaccharides on airway inflammation of lungs in an asthma mouse model

**DOI:** 10.1186/s40248-015-0017-0

**Published:** 2015-06-11

**Authors:** Rongjun Lin, Xiaomei Liu, Yan Meng, Mei Xu, Jianping Guo

**Affiliations:** Department of Pediatrics, The Affiliated Hospital of Qingdao University Medical College, Qingdao, 266003 China; Department of Pediatrics, People’s hospital of Zoucheng city, Jining, 273500 China; Department of Pediatrics, People’s Hospital of Central District, Zaozhuang, 277101 China; Department of Pediatrics, Women and Children’s Hospital of Qingdao, Qingdao, 266011 China

**Keywords:** Asthma, Allergy, Inflammation, Sulfated polysaccharides, Immunomodulation

## Abstract

**Background:**

Asthma is a serious chronic inflammatory disease affecting 300 million people worldwide. This aim of this study to investigate the anti-inflammatory and anti-asthmatic effects of *Laminaria japonica* extract in the ovalbumin (OVA)-induced mouse asthma model.

**Methods:**

A mouse asthma model was established in SPF Kunming mice by OVA-sensitization followed by inhalation of aerosol allergen for two weeks. *Laminaria japonica* polysaccharides (LJPS) were given by gavage feeding at 50 mg/kg/day during OVA inhalation challenge period, and their effect on asthma was compared with the standard treatment of Budesonide inhalation. The total inflammatory cells and eosinophils in bronchoalveolar lavage fluid (BALF) were determined. Histopathological changes in lung tissue were studied and scored to determine the degree of inflammation. Levels of IL-12, IL-13, and TGF-β1 in BALF as well as serum levels of IgE were measured. Expressions of IL-12, IL-13, and TGF-β1 in lung tissues were assessed.

**Results:**

Highly inflammatory lungs infiltrated with significant increased eosinophils were observed in OVA-induced asthmatic mice. The OVA treated mice presented with a lower level of IL-12 and higher levels of IL-13 and TGF-β1 in BALF and lung tissues, as well as an increased level of the serum IgE. Treatment with LJPS (Group B) significantly decreased the numbers of eosinophils in the BALF (*P* < 0.05) and alleviated lung inflammation compared to the untreated asthma mice (Group A). It also reduced the serum IgE levels, increased expression of IL-12, and decreased the expression of IL-13 and TGF-β1 in BALF and lung (Both *P* < 0.05) compared with the group A.

**Conclusions:**

LJPS can significantly inhibit airway inflammation of asthmatic mice, adjust the balance of cytokines, and improve the pulmonary histopathological condition. Our data suggested that LJPS might be a potential therapeutic reagent for allergic asthma.

## Background

Asthma is a serious chronic inflammatory disease characterized by reversible lung airway obstruction [1]. The incidence of asthma has been on the rise globally. It is estimated that asthma affects 300 million people and contributes to 250,000 annual deaths worldwide. Pathologically, patients are found to have bronchial mucosal thickening by edema, bronchial wall remodeling, mucus overproduction, and eosinophil infiltration [2].

The general approach of current asthma therapy is to control the inflammatory response in the airways using corticosteroids. The commonly used treatments for asthma include bronchodilators of long-acting beta agonists and muscarinic antagonists or anti-inflammatory drugs such as corticosteroids. While corticosteroids are very effective in controlling the symptoms, there are 5–10 % of asthma patients who are glucocorticoid insensitive. The side effects of corticosteroids have been the major concern, and the therapeutic effects are far from fully satisfactory [3, 4]. Despite the fact that significant progresses have been achieved in the field of asthma research, new approaches for asthma treatment are necessary to ensure alleviation of symptoms in all patients.

Traditional Chinese medicines are gaining recognition for their therapeutic effect in alleviating asthmatic symptoms. *Laminaria japonica* polysaccharides (LJPS), an extracted mixture containing alginic acids, laminarans and sulfated polysaccharides (fucoidans) from the brown sea algae *Laminaria japonica,* have been investigated in various studies for its biological function. These polysaccharides have been implicated in a number of functions, including antioxidant and free radicals scavenging, anti-inflammatory, antitumor, reducing blood lipids, and anti-diabetes [5–7]. In this study, we further analyzed the anti-inflammatory properties of LJPS in a mouse model of allergic asthma that mimicked asthma in patients. Our data suggested that LJPS suppressed inflammation and had a therapeutic potential for asthma.

## Material and methods

### Mice

Female SPF Kunming mice aged 6–8 weeks (18–22 g) were purchased from the Experimental Animal Center. The protocols of animal experiments were reviewed and approved by the Institute of Animal Care and Use Committee of the Qingdao Medical University. The animals were maintained in the clean barrier animal facilities under specific pathogen-free conditions. All animals were euthanized by CO_2_ chamber.

### Ovalbumin (OVA)-induced asthma mouse model and treatment procedures

An OVA-induced asthma mouse model was established as described in previous studies with modifications [8]. Started from 21 days after initial administration of OVA (Sigma-Aldrich, USA), mice were randomly grouped and treated differently for two weeks as the following groups: A, untreated group; B, LJPS-treated groups; and C, Budesonide-treated group. Control animal (group D) was sensitized and challenged with PBS without OVA. Each group was set with 10 mice. No treatment was applied to control mice. LJPS was given at 50 mg/kg in normal saline (Jinan, China) per day through gavage feeding. Budesonide (AstraZeneca, UK) was given at 200 pg in 4 ml saline by inhalation daily. Mice were challenged 30 min daily for two weeks by an inhalation of 2 % OVA through an air aerification inhaler in an atomization inhalation chamber.

### Preparation of LJPS

*Laminaria japonica* was harvested in Rongcheng, Shandong, China. The *Laminaria japonica* was dried and pulverized to powder. Dry powder (50 g) was mixed with 1000 ml of distilled water with 0.02 % (w/w) cellulose enzyme, 0.05 % (w/w) papain, and 0.05 % (w/w) neutral protein enzyme, and then incubated at 70 °C for 6 h followed by another 12 h incubation at room temperature after adjusting pH to 10.0. The mixture was centrifuged at 500 g for 15 min and supernatant (A) was collected. 500 ml of 10 % HCl was added to the pellet, incubated at room temperature for another 4 h, and centrifuged again (500 g, 15 min) and supernatant (B) was collected. The supernatant (B) was combined supernatants (A) and precipitated with 80 % ethanol. The precipitate was subjected to DEAE cellulose column chromatography. The final product was measured as glucose (5.6 %), mannose (22.10 %), rhamnose (7.0 %), galactose (8.0 %), and xylose (56.2 %). The molecular weight of polysaccharide was 67 kDa.

### BALF collection and cell count

24 h after the last OVA challenge, the animal was anesthetized with an *i.p.* injection of 10 % chloral hydrate (Sigma-Aldrich, USA) in PBS, and then the trachea was cannulated. Bronchoalveolar lavage was performed by flushing 0.3 ml of PBS into the trachea through the cannula, and the cells in the lung were collected in bronchoalveolar lavage fluid (BALF). The process was repeated three times.

The BALF was immediately centrifuged at 3000 RPM/min for 5 min at 4 °C. The supernatant was stored at−20 °C. Pellets were re-suspended in 50 μl PBS. Total cell counts were determined, and differential cell counts were determined according to standard morphological criteria. The number of eosinophils in every 200 inflammatory cells in BALF was calculated.

### Histological examination of lung tissues

Histological examination was performed by Hematoxylin and Eosin (HE) staining as described previously with minor modifications [8]. Briefly, animal was euthanized 24 h after the last OVA challenge or PBS inhalation., The middle lobe of the right lung was excised, fixed in 4 % formalin, and embedded in paraffin after the left lung had been lavaged. Sections of 5 μm were de-paraffinized and hydrated, and then stained with HE or immunohistochemistry staining.

Histopathological assessment (light microscopy) on randomly selected sections was performed by a pathologist who was blinded from the experiment. Inflammatory responses were graded using a semi-quantitative scale of 0–5 for epithelial damage, perivascular eosinophilia, peribroncholar eosinophilia, and edema as described previously [2].

### ELISA

Quantitative assessments of IL-12, IL-13 and TGF-β1 in BALF were conducted using enzyme linked immunosorbent assay (ELISA) kits (R&D Systems Inc. USA.), whereas serum IgE levels were assessed using ELISA kit (Beijing Zhongshan Golden Bridge, China) according to the manufacturers’ instructions.

### Immunohistochemistry for IL-12, IL-13 and TGF-β1

Sections of lung tissue were studied by immunohistochemistry methods to detect and localize IL-12, IL-13 and TGF-β1 protein expression using corresponding rabbit polyclonal antibodies (Beijing Golden Bridge Co., China). Chromogen 3,3-diaminobenzidine (Beijing Golden Bridge Co., China) was used as substrate, and sections counterstained with PBS instead of primary antibody were used as negative control.

### Statistical analysis

Statistical analysis was performed with the SPSS 17.0 software (IBM, USA). Data were expressed as mean ± SEM (standard error of the mean). Differences between the means of two groups were determined by one-way ANOVA. Paired t-tests were used to assess the statistical differences between the paired samples. In all cases, if a p value was less than 0.05, it would be considered statistically significant.

## Results

### Manifestation of allergen-induced asthmatic mice

Mice challenged with OVA inhalation showed obvious signs of sickness, including sneezes, nose rubbing, breathing deeply and fast, lip and eye cyanosis, ruffled fur, forelimb shrinkage lift, stooping, irritability, and other various degrees of asthma immediate responses. These symptoms persisted in mice treated with PBS (Group A); however, in mice treated with either LJPS (Group B) or Budesonide (Group C) alleviated symptoms were observed. The mice in the control group (Group D) did not show any of the above symptoms.

### Anti-inflammatory effects of LJPS in allergic asthma

**Fig. 1:**
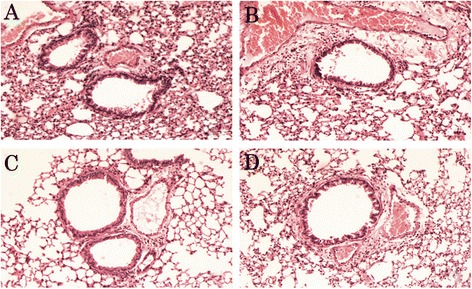
**a**–**d**. Histopathological changes of the experimental mice with various treatments. Lung sections of the OVA-induced asthmatic mice treated with PBS (**a**), LJPS (**b**), Budesonide (**c**), and PBS treated control mice (**d**) were stained with Hematoxylin and eosin

Significant eosinophil infiltration was observed by HE staining of sections from OVA-challenged mice in both perivascular and peribronchial areas (Fig. 1a). The inflammation was greatly alleviated when treated with either LJPS or Budesonide (Fig. 1, b and c, Group B and C), whereas no eosinophil infiltration was found in the control group (Fig. 1d, Group D). The pathological score of lung tissue was graded according to the degree of perivascular eosinophilia, peribroncholar eosinophilia, epithelial damage, and edema with a semi-quantitative scale (Table 1). The pathological scores were significantly higher for in OVA challenged mice than the control mice (*P* < 0.05 by paired t test). Treatment with either LJPS (Group B) or Budesonide (Group C) was able to bring down the pathological scores of the lung tissues in these asthmatic mice (*P* < 0.05 in comparison of OVA challenged group by paired t test).

**Table 1:** Histopathological analysis of lung tissue sections

Group	#	Eosinophil infiltration	Edema	Airway epithelial
A	10	4 (1–5)	4 (2–5)	4 (2–5)
B	10	2 (1–4)	3 (1–3)	1 (0–2)
C	10	1 (0–1)	1 (0–1)	0 (0–0)
D	10	1 (0–1)	0 (0–0)	0 (0–0)

Group A, B, C were challenged with OVA. A, treated with PBS; B, treated with LJPS; C, treated with Budesonide; D, control group challenged by PBS. Data were expressed as median (range)

The inflammatory cell and eosinophil count in BALF further demonstrated that LJPS treatment could reduce the OVA-induced hyper-responsiveness similar to what was observed with Budesonide treatment. BALF was collected 24 h after the last OVA or PBS aerosol inhalation. As shown in Table 2, the total inflammatory cell number in BALF from OVA challenged mice was significantly elevated compared to that from mice of PBS inhalation. Both Budesonide (Group C) and LJPS (Group B) treatment significantly reduced the inflammatory cell count in BALF for the asthmatic mice (*P* < 0.05 by ANOVA test).

**Table 2:** Infiltrated inflammatory cell count in BALF (×10^5^/ml)

Groups	#	WBC	Eosinophils
A	10	23.09 ± 3.09	3.42 ± .42
B	10	11.51 ± 1.51*	0.38 ± .381*
C	10	9.13 ± .131	0.09 ± 0.07
D	10	6.84 ± .841	0.07 ± .071

Group A, B, C were challenged with OVA followed by treatment with PBS (A), LJPS (B), or Budesonide (C) respectively. Group D was the control group challenged by PBS. Data were expressed as mean ± SEM. *P* < 0.05 in groups B, C, and D vs group A

*,*P* < 0.05 in group B vs group C

### Serum IgE levels

**Fig. 2:**
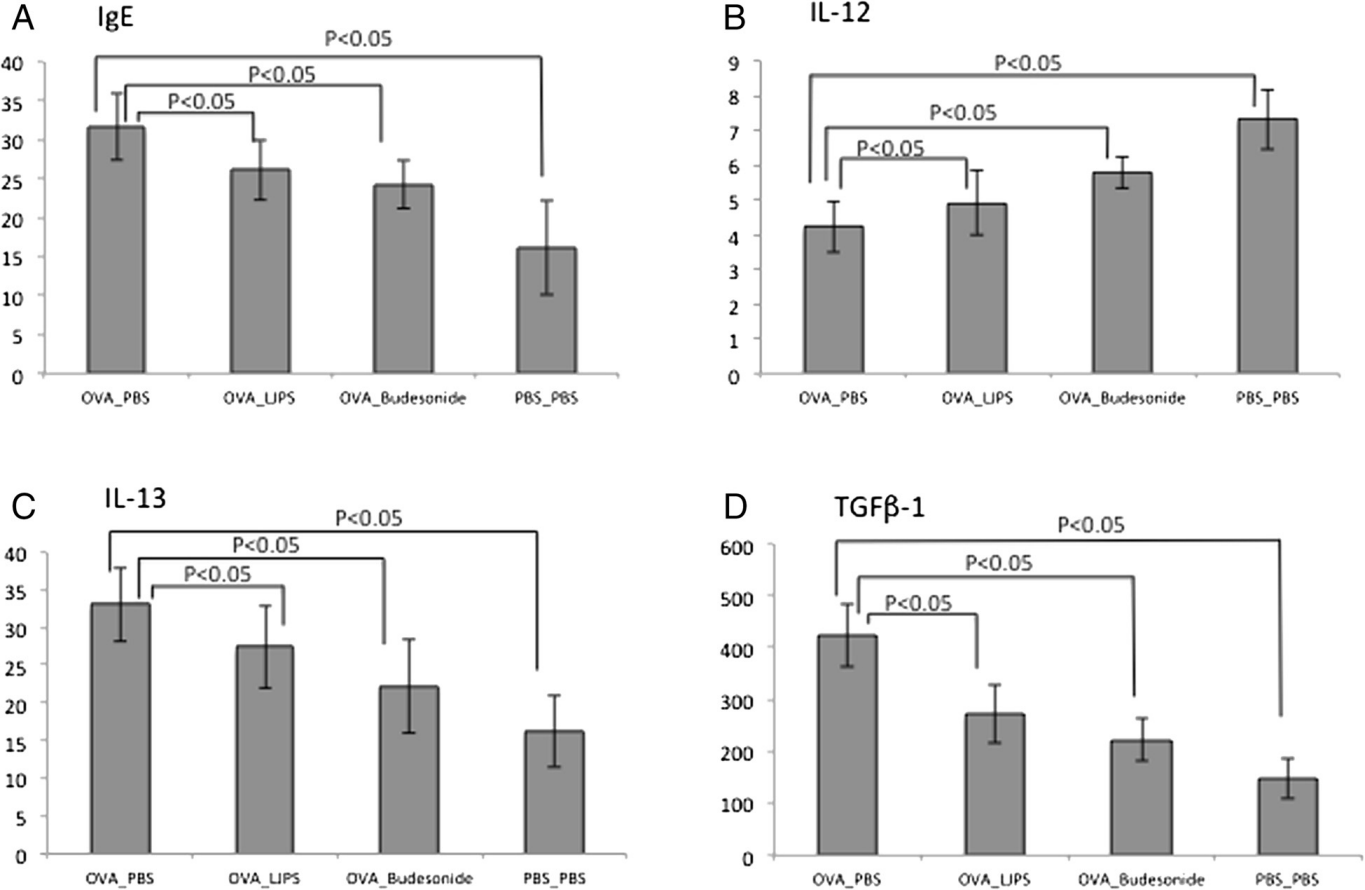
**a**–**d**. Immune responses of OVA-challenged mice upon various treatments. Serum IgE levels (**a**), and cytokine levels of IL-12 (**b**) (**c**) IL-13 TGF-β1 (**d**) in BALF of the OVA-induced asthmatic mice underwent various treatments were assessed and statistical analysis was performed

As shown in Fig. 2a, OVA-challenged mice had a significantly higher serum IgE level of 31.65 ng/ml, while the IgE level from control mice was 16.19 ng/ml (*P* < 0.05 by ANOVA test). LJPS treatment (Group B) was able to reduce the IgE level to 26.07 ng/ml, (*P* < 0.05 comparing to OVA challenged mice by ANOVA test), which was similar to the effect of Budesonide treatment (24.25 ng/ml, Group C).

### Cytokine in BALF and expression in lung tissue

We investigated whether LJPS can reduce inflammation by similar down-regulation of the Th2 cytokines. Cytokine in the BALF was measured by ELISA. As shown in Fig. 2, b-d, the level of IL-12 was greatly reduced in OVA-challenged mice compared to the control, while IL-13 and TGF-β1 were greatly increased. All these changes were reversed with the treatment of LJPS or Budesonide. We observed significantly increased IL-12 level as well as reduced levels of IL-13 and TGF-β1 in BALF from LJPS or Budesonide treated mice (*P* < 0.05).

**Fig. 3:**
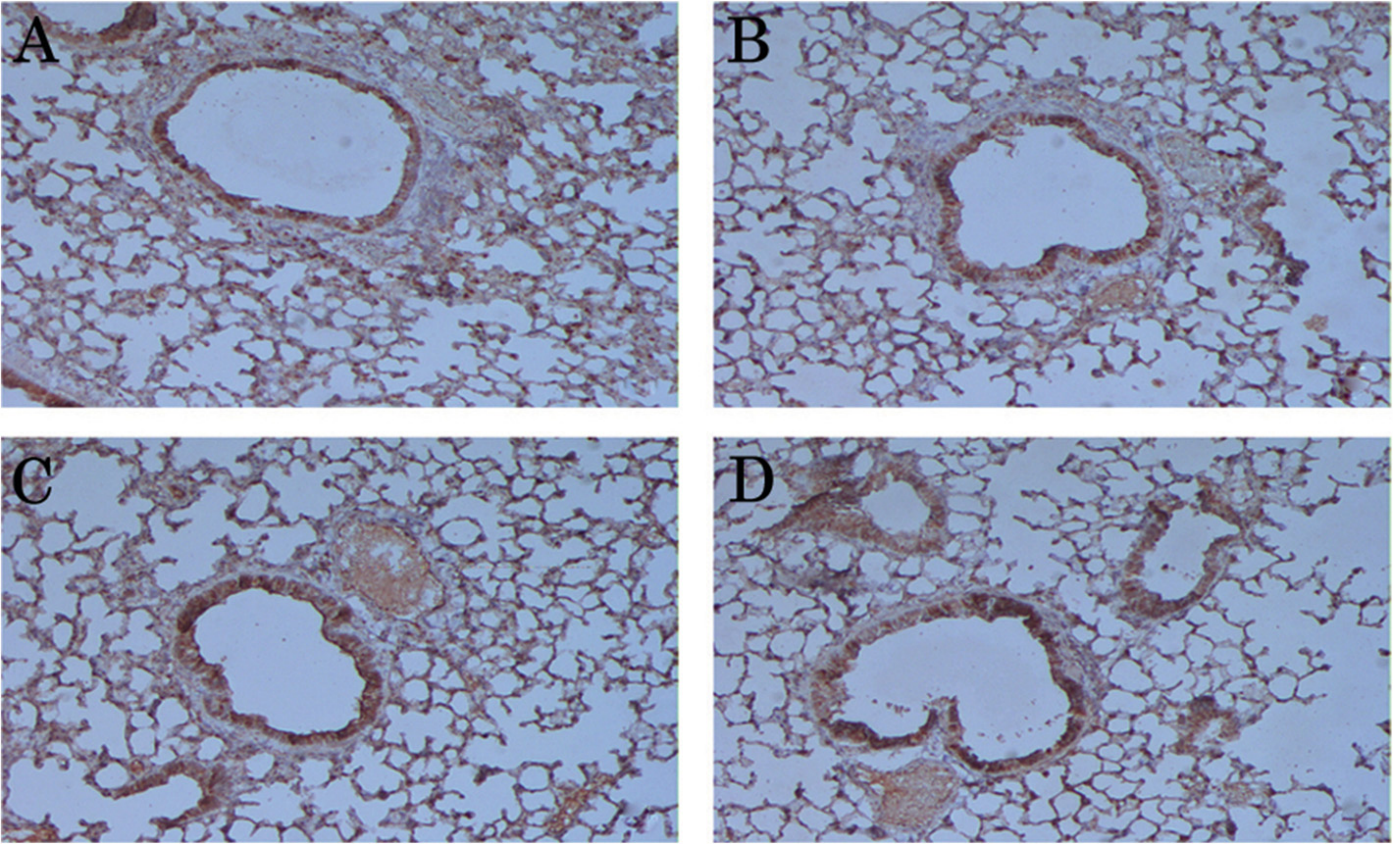
**a**–**d**. Expression and localization of IL-12 in the lung tissue. IL-12 protein was detected by Immunohistochemistry in tissue sections from OVA-induced asthmatic mice treated with PBS (**a**), LJPS (**b**), and Budesonide (**c**), as well as PBS-treated control mice (**d**). The cytoplasm and nucleus in positive cells appeared brown yellow fine particles in lung tissues

**Fig. 4:**
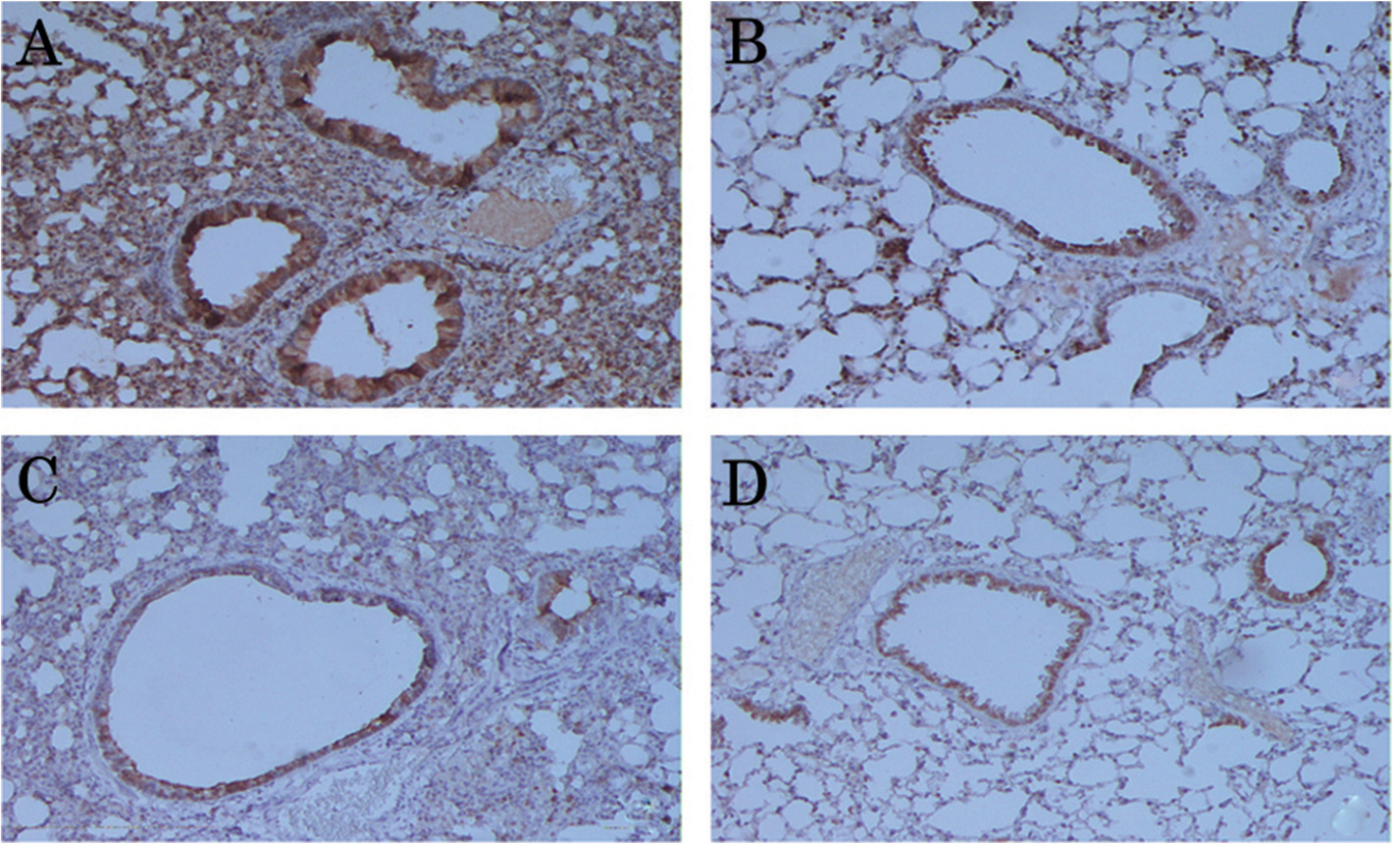
**a**–**d**. Expression and localization of IL-13 in the lung tissue. IL-13 protein was detected by Immunohistochemistry in tissue sections from OVA-induced asthmatic mice treated with PBS (**a**), LJPS (**b**), and Budesonide (**c**), as well as PBS-treated control mice (**d**). The cytoplasm and nucleus in positive cells appeared brown yellow fine particles in lung tissues

We further studied the localization of these cytokines *in situ* in the formalin-fixed, paraffin-embedded lung tissue sections. IL-12 expression was reduced in the lungs of asthmatic mice compared to those of the un-induced control; expression was mostly localized in the cytoplasm of the bronchial epithelial cells (Fig. 3a and d). We observed higher expression of IL-12 in both LJPS and Budesonide-treated asthmatic mice than in the non-treated asthmatic mice (Fig. 3b and c vs. a). On the other hand, the levels of IL-13 were significantly elevated in the lung tissues of OVA-induced asthmatic mice (Fig. 4a) compared to the PBS control mice (Fig. 4d). LJPS treatment led to the down-regulation of IL-13 expression, similar to the treatment of Budesonide (Fig. 4b and c). IL-13 was also seen mostly in the cytoplasm of bronchial epithelial cells.

**Fig. 5:**
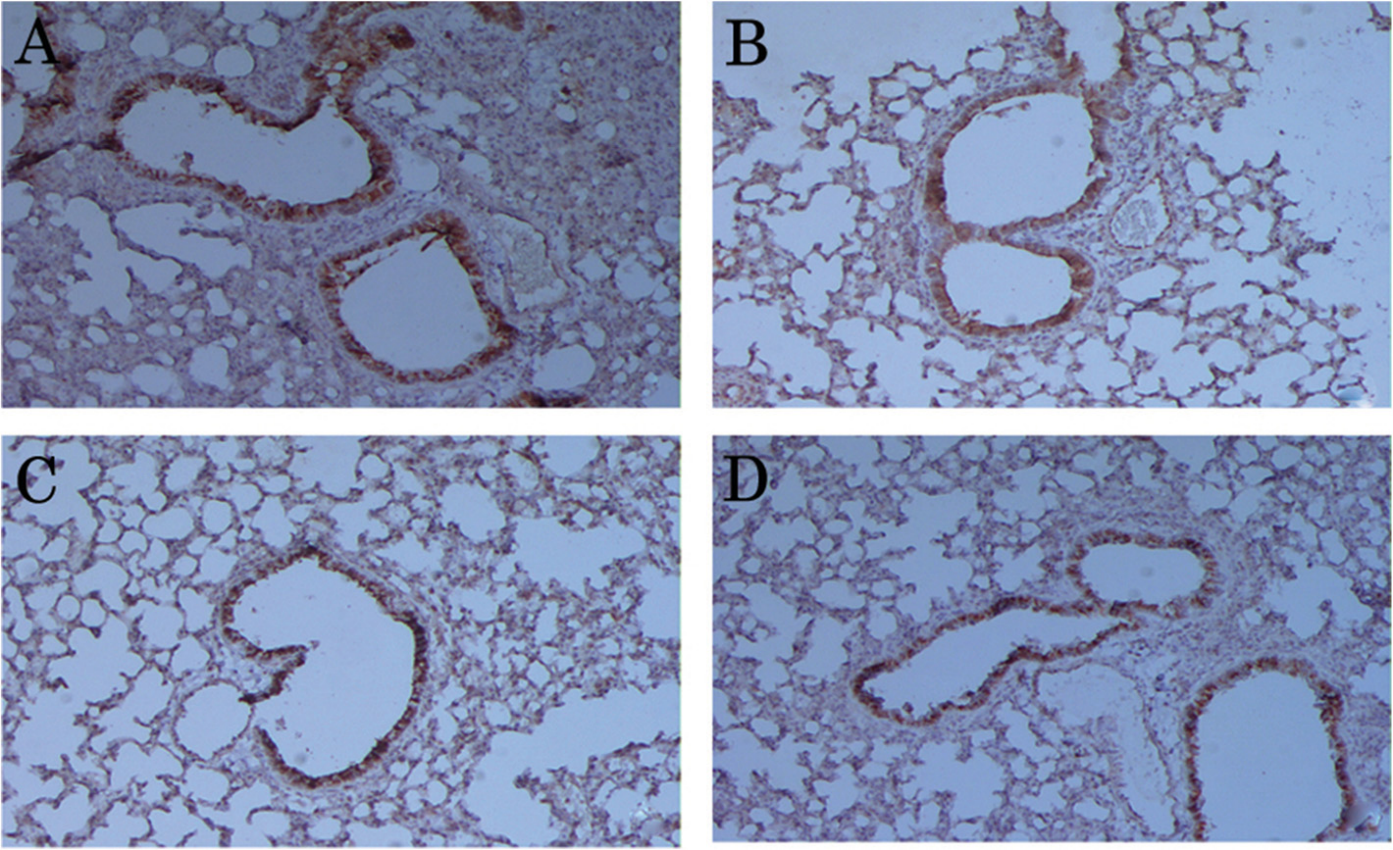
**a**–**d**. Expression and localization of TGF-β1 in the lung tissue. TGF-β1 protein was detected by Immunohistochemistry in tissue sections from OVA-induced asthmatic mice treated with PBS (**a**), LJPS (**b**), and Budesonide (**c**), as well as PBS-treated control mice (**d**). The cytoplasm and nucleus in positive cells appeared brown yellow fine particles in lung tissues

TGF-β1 expression was markedly increased in the lung tissues of OVA-induced asthmatic mice (Fig. 5a), reduced in both LJPS and Budesonide treated mice (Fig. 5b and c), while little or no expression was detected in the lung tissue of PBS-sensitized normal mice (Fig. 5d). Strong cytoplasmic staining was observed in bronchial epithelial cells, connective tissue of the lamina propria and adventitia, smooth muscle cells, and inflammatory cells (Fig. 5a).

## Discussion

Inflammatory responses were assessed *in situ* by histologic evaluation of lung tissue sections as well as cell counts in the retrieved BALF. The effect of LJPS on lung inflammation in the asthmatic mice was compared to that of Budesonide, one of the most effective anti-inflammatory drugs used in allergic asthma treatment.

OVA-induced asthmatic model is a well-established animal model for studying the mechanism of asthma and therapeutic effects of drugs. Upon OVA challenge, the mice exhibited similar symptoms to those found in patients with chronic asthma, including different degrees of immediate asthma reactions, eosinophil infiltration into the lung interstitium and BALF, as well as epithelial damage in the lung. Therefore, it provided an ideal model to mimic clinical reality.

Polysaccharides are considered as T-cell independent antigens that only elicit humoral immune response in general. However, recent studies pointed out that certain polysaccharides could be strong immune modulators. Several clinical studies have revealed its effect on increasing the number of cytotoxic T cells and the phagocytic capacity of monocytes, as well as reducing inflammatory cytokines [9, 10]. We employed LJPS in the treatment of experimental asthma and found that it reduced airway inflammation in the mouse asthma model. We found that oral treatment of LJPS in OVA-induced asthmatic mice exhibited symptom alleviation similar to treatment with Budesonide. The inflammation in the lung tissue was reduced, as were the infiltrating inflammatory cells, including eosinophils.

It has been known that the imbalance of Th1 and Th2 cytokines is responsible for the pathogenesis of asthma. Th2 cytokines were found to be up-regulated in asthma that led to over production of TGF-β1, while Th1 cytokine expression was suppressed. Infiltration of mononuclear cells, mostly CD4 T helper type 2 cells (Th2) and eosinophils, in the airway wall underlines the major pathogenesis of asthma. A dominant Th2 response characterized by overproduction of Th2-type cytokines, an elevated level of IgE, eosinophil recruitment, and mast cell activation, has been directly linked to asthma severity. Th2-type cytokines IL-4, IL-5, and IL-13 have been implicated in promoting allergic responses in asthma [11, 12]. These cytokines, IL-13 in particular, play critical roles in the human allergic responses. IL-13 has been shown to potentiate IgE production, upregulate the expression of adhesion molecules, as well as induce mucus hyper-secretion and airway hyper-responsive [13, 14]. Th1-type cytokine IL-12, on the other hand, is involved in reducing allergen-specific IgE and airway eosinophilia [15, 16]. Asthmatic patients had significantly reduced levels of IL-12 in both peripheral blood and airway biopsy specimens compared with their healthy counterparts [17]. IL-12 is known to promote T cell differentiation toward a Th1-mediated response while suppressing the expansion and differentiation of Th2 cells [18]. Glucocorticoid treatment typically suppresses Th2-type cytokines, and reduces eosinophilic inflammation. LJPS treatment, similarly to Budesonide, reduced the levels of IL-13 in both BALF and lung tissue *in situ* in the asthmatic mice, and restored IL-12 expression in lung, therefore to re-establish the balance of Th1 and Th2 immunity. As a consequence, the serum IgE levels were also reduced after LJPS treatment.

Structural changes, namely airway remodeling, occur in the airways of asthmatic patients. These changes include goblet cell hyperplasia, increased thickness of subepithelial basement membrane, increased mass and size of airway smooth muscle (ASM), and fibrosis. TGF-β, a potent profibrotic cytokine, is a major player in regulating airway remodeling [19, 20]. TGF-β induces the expression and release of profibrotic and proinflammatory cytokines in fibroblasts and ASM cells. It can also serve as a chemo-attractant for monocytes, fibroblasts, and mast cells. The level of TGF-β was found to increase in both mild and severe asthmatics, and its level correlates with basement membrane thickness [21, 22]. Eosinophils, macrophages, and fibroblasts were reported as the main sources of TGF-β1 [22–24]. Scherf *et al*. showed that TGF-β1 could increase levels of the Th2 cytokines, including IL-4, IL-5, and IL-13, resulting in an increased inflammatory response in the lung [25]. We found in our study that TGF-β1 levels increased in both the BALF and lung tissue sections of the OVA-induced asthmatic mice. LJPS treatment led to an almost 40 % reduction of TGF-β1 levels in these mice, similar to the reduction caused by Budesonide treatment. The reduction of TGF-β1 level clearly correlates to the histopathology seen in these mice, and to the reduction of eosinophil infiltrations seen after treatment. Previously, Xiong *et al.* [26] reported that a Chinese herbal medicine, (±)-Praeruptorin A, was able to reduce the expression of TGF-β1 in an OVA-sensitized asthma model. In their study, the reduction of TGF-β1 was also concurrent with the improvement of lung inflammation. We hypothesize that LJPS may inhibit mouse airway inflammation through reduction of the expression of TGF-β1.

Polysaccharides are usually recognized by TLR4 in inducing native immune response. Study has shown that sulfated polysaccharides from sea brown algae can interact with TLR2 and TLR4 in vitro using transfected HEK cells [27]. It has also been shown that fucose-containing sulfated polysaccharides, may induce dendritic cell maturation, promoting IL-12, TNFα production [28, 29]. Another study showed that the dietary fucoidan activated the T-cell mediated cytotoxic activity and NK cell function [30]. It is possible that LJPS modulates the inflammation in the asthmatic response through cytokines produced by activated native immunity. Future studies on the effect of LJPS on macrophages and NK cell activities will provide clues to its mechanisms.

The study was limited by the use of the OVA-induced asthma mouse model, which defined the application potential of the LJPS in allergic asthma. For non-allergic asthma, the therapeutic efficacy of LJPS needs to be further studied.

## Conclusion

LJPS could be used as an allergic asthma treatment in a mouse model. The administration of LJPS alleviated asthma symptoms, reduced airway inflammation by reducing Th2 cytokines and increasing a Th1 cytokine, as well as reducing the expression of TGF-β1 in lung. These results suggested that LJPS has a significant anti-inflammatory effect on allergen-induced lung inflammation in this asthma mouse model, providing a strategy for adoptive immunotherapy for asthma treatment without immune suppression.
